# Mitochondrial genome diversity and evolution in Branchiopoda (Crustacea)

**DOI:** 10.1186/s40851-019-0131-5

**Published:** 2019-05-27

**Authors:** Andrea Luchetti, Giobbe Forni, Alyza M. Skaist, Sarah J. Wheelan, Barbara Mantovani

**Affiliations:** 1 Department of Biological, Geological and Environmental Sciences, University of Bologna, via Selmi 3, 40126 Bolgna, Italy; 2 Department of Oncology, Sidney Kimmel Comprehensive Cancer Center, The Johns Hopkins University School of Medicine, Baltimore, MD 21205 USA

**Keywords:** Branchiopoda, Mitochondrial genomics, Mitochondrial unequal recombination, Notostraca, Nucleotide compositional bias, Nucleotide substitution rate

## Abstract

**Background:**

The crustacean class Branchiopoda includes fairy shrimps, clam shrimps, tadpole shrimps, and water fleas. Branchiopods, which are well known for their great variety of reproductive strategies, date back to the Cambrian and extant taxa can be mainly found in freshwater habitats, also including ephemeral ponds. Mitochondrial genomes of the notostracan taxa *Lepidurus apus lubbocki* (Italy), *L. arcticus* (Iceland) and *Triops cancriformis* (an Italian and a Spanish population) are here characterized for the first time and analyzed together with available branchiopod mitogenomes.

**Results:**

Overall, branchiopod mitogenomes share the basic structure congruent with the ancestral Pancrustacea model. On the other hand, rearrangements involving tRNAs and the control region are observed among analyzed taxa. Remarkably, an unassigned region in the *L. apus lubbocki* mitogenome showed a chimeric structure, likely resulting from a non-homologous recombination event between the two flanking *trnC* and *trnY* genes. Notably, Anostraca and Onychocaudata mitogenomes showed increased GC content compared to both Notostraca and the common ancestor, and a significantly higher substitution rate, which does not correlate with selective pressures, as suggested by dN/dS values.

**Conclusions:**

Branchiopod mitogenomes appear rather well-conserved, although gene rearrangements have occurred. For the first time, it is reported a putative non-homologous recombination event involving a mitogenome, which produced a pseudogenic tRNA sequence. In addition, in line with data in the literature, we explain the higher substitution rate of Anostraca and Onychocaudata with the inferred GC substitution bias that occurred during their evolution.

**Electronic supplementary material:**

The online version of this article (10.1186/s40851-019-0131-5) contains supplementary material, which is available to authorized users.

## Background

The class Branchiopoda is a small but highly diverse group of crustaceans, distributed on all continents and inhabiting both marine and inland waters, including hypersaline lakes and ephemeral or freezing ponds [1–4]. The origin of branchiopods dates back to the Middle Cambrian [5] and the class includes forms retaining remarkably conserved ancestral characters, like the so-called “living fossils” genera *Triops* and *Lepidurus* (tadpole shrimps of the order Notostraca).

The class taxonomy has been extensively revised during the last decades and it is presently considered to include four orders: Anostraca (fairy shrimps), Notostraca (tadpole shrimps), Laevicaudata (clam shrimps), and Onychocaudata (Spinicaudata [clam shrimps] + Cyclestherida [clam shrimps] + Cladocera [water fleas]) [3]. A clear phylogenetic picture of within-Branchiopoda relationships emerged recently with phylogenomic analyses, nicely reconciling with morphological hypotheses. These analyses supported Anostraca as the sister group to Phyllopoda, a clade including all other branchiopods, in which Notostraca are the sister group to Diplostraca (=Laevicaudata + Onychocaudata) [4, 6–8].

The relevance of mitochondrial genomics analyses encompasses many fields of biology, ranging from comparative biology studies, to medically related questions and cellular molecular biology [9]. On the other hand, the greatest contribution of mitogenomics has probably been in the fields of molecular phylogeny and evolution [10, 11]. The animal mitochondrial genome is a relatively simple molecule, with a general structure that is strongly conserved across the animal kingdom. It is circular, approximately 16,000 bp long, with 13 protein coding genes (PCGs), two rRNA genes, 22 tRNA genes, and a non-coding region of variable length, known as control region [10]. Non-bilaterian animal taxa show remarkable exceptions to this organization [12], with instances of variation in length and gene content. Gene rearrangements are also present in bilaterian taxa (mainly duplications and translocations), with the remarkable instance of the phylum Mollusca [13, 14].

Although generally well-conserved, arthropod mitogenomes also exhibit some variations, ranging from rearrangements of the gene order in some crustaceans [15, 16] and insects [17], to duplications of one or a few tRNA genes [18–20] or of the control region [17]. The study of mitogenomic gene arrangement uncovered a higher plasticity of the organization of this molecule than previously thought [13]. Moreover, it also serves as a tool for both improving phylogenetic estimation [11] and understanding mechanisms responsible for mitogenome structural variation [21].

In this article, we report on the sequencing and analysis of four mitogenomes from three taxa of Notostraca. In particular we obtained, for the first time, the whole mitochondrial genomes of two *Lepidurus* taxa: the Mediterranean form *L. apus lubbocki* and the arctic species *L. arcticus* [22]. Moreover, although the *Triops cancriformis* mitogenome has previously been sequenced from a sample collected in an invasive population in Japan [23], we here present mitogenomes from two distinct, native populations of *T. cancriformis* with different reproductive modes: a parthenogenetic population (collected in Italy) and a gonochoric one (from Spain) [24]. Finally, newly obtained mitogenomes are also analyzed with all currently available branchiopod mitogenomes in order to draw insights into mitogenome evolution in this class of crustaceans.

## Methods

Total DNA was extracted from individual specimens using the DNA extraction kit (STRATEC), after dissection for gut removal. Whole genome sequencing has been carried out on Illumina HiSeq2000 (Johns Hopkins University Experimental and Computational Genomics Core, SKCCC, USA) on parthenogenetic *T. cancriformis* sample (paired end, 2 × 100 bp; insert size = 150 bp; Luchetti, unpublished data), and on HiSeqX platform (Macrogen Inc., South Korea) on bisexual *T. cancriformis* and *Lepidurus* taxa (paired end, 2 × 150 bp; insert size = 350 bp) [25]. Mitochondrial genomes were de novo assembled from a random subset of 1 million read pairs per sample using the SPAdes v. 3.11.1 assembler with default parameters [26]. Coverage was calculated by mapping raw reads with Bowtie2 [27] and analyzing the output with SAMtools [28]. Annotation was de novo performed using the MYTOS pipeline [29] and manually corrected by similarity with the published *T. cancriformis* mitochondrial genome (GenBank accession number: AB084514). Genes for tRNAs were confirmed by means of ARWEN v. 1.2.3 [30].

A selection of sequenced mitochondrial genomes of branchiopods, and two outgroup insect sequences, were downloaded from GenBank (Table 1). Alignments of PCGs and of rRNA genes were performed with MAFFT v. 7.205 [47], using the automatic detection of parameter set. Alignments of PCGs were carried out considering the amino acid translation to correctly align codons. Alignments for ribosomal RNA genes (*rrnL* and *rrnS*) were filtered using Gblocks v. 0.91b [48], with options for less stringent selection (min. Number of sequences for a conserved position = 22; min. Number of sequences for a flanking position = 22; max. Number of contiguous non-conserved positions = 8; min. Length of a block = 5; allowed gap positions = with half). All alignments were concatenated, and the best partition scheme and substitution models were estimated using Partition Finder v. 1.1.1 [49]. The best partition schemes and best-fitting substitution models are reported in Additional file 3: Table S1. Maximum-likelihood trees were obtained using RAxML v. 8.2 [50], using the rapid bootstrap option with 500 replicates for nodal support. Bayesian Inferences were obtained with MrBayes v. 3.2.3 [51]. Two tree searches were run for 10,000,000 generations and sampling every 1000 trees. Convergence was assessed by average standard deviation of split frequencies < 0.01. The BI tree was summarized after a conservative *burnin* = 25%.

**Table 1:** Samples obtained from Genbank for comparative and phylogenetic analyses

Taxonomy			Species	Genbank acc. Nos.	Reference
Crustacea	Branchiopoda	Anostraca	*Artemia franciscana*	X69067	[31]
Crustacea	Branchiopoda	Anostraca	*A. tibetiana* 1	JQ975177	[32]
Crustacea	Branchiopoda	Anostraca	*A. tibetiana* 2	JQ975178	[32]
Crustacea	Branchiopoda	Anostraca	*A. urmiana*	JQ975176	[32]
Crustacea	Branchiopoda	Anostraca	*Phallocryptus tserensodnomi*	KP273592	[33]
Crustacea	Branchiopoda	Anostraca	*Streptocephalus sirindhornae*	KP273593	[34]
Crustacea	Branchiopoda	Cladocera	*Daphnia carinata*	KP721459	[35]
Crustacea	Branchiopoda	Cladocera	*D. magna*	KP296147	[36]
Crustacea	Branchiopoda	Cladocera	*D. pulex*	AF117817	[37]
Crustacea	Branchiopoda	Cladocera	*D. pulex* (China)	KT003819	[38]
Crustacea	Branchiopoda	Cladocera	*D. galeata*	LC177070	[39]
Crustacea	Branchiopoda	Cladocera	*Diaphanosoma dubium*	MG428405	[40]
Crustacea	Branchiopoda	Spinicaudata	*Limnadia lenticularis*	MH618637	[41]
Crustacea	Branchiopoda	Notostraca	*Triops australiensis*	LK391946	[42]
Crustacea	Branchiopoda	Notostraca	*T. cancriformis* (Japan)	AB084514	[23]
Crustacea	Branchiopoda	Notostraca	*T. longicaudatus* 1	AY639934	[43]
Crustacea	Branchiopoda	Notostraca	*T. longicaudatus* 2	GU475465	[44]
Crustacea	Branchiopoda	Notostraca	*T. longicaudatus* “l”	KM516710	[45]
Crustacea	Branchiopoda	Notostraca	*T. longicaudatus* “s”	KM516711	[45]
Crustacea	Branchiopoda	Notostraca	*T. newberry*	KM516712	[45]
Hexapoda	Insecta	Thysanura	*Thermobia domestica*	AY639935	[43]
Hexapoda	Insecta	Isoptera	*Reticulitermes flaviceps*	KX712090	[46]

The hypothesis of constancy of substitution rate throughout the tree was tested by comparing the −*lnL* of tree with and without strict clock constraint by means of likelihood ratio test, as implemented in MEGA v.7 [52]. A time tree was also calculated by means of BEAST v. 1.8 [53]: two independent chains were run for 60,000,000 generations each and trees sampled every 1000 generations. Mixing and convergence of the two runs was reached when effective sample size of each parameter > 200. The maximum-clade-credibility tree was determined after discarding the first 10% of the trees obtained (*burnin*). Clock model was set to a lognormal uncorrelated relaxed clock, and a birth-death speciation model was assumed. Calibrations were set on crown groups, using a lognormal probability distribution, based on detailed fossils description given in Wolfe et al. [54]: Notostraca were dated using *Chenops yixianensis* fossil taxon; minimum hard bound = 121.8 Mya, maximum soft bound = 521 Mya; Cladocera were dated using *Smirnovidaphnia smirnovi* fossil taxon; minimum hard bound = 173.1 Mya, maximum soft bound = 521 Mya; Anostraca were dated using *Palaeochirocephalus rasnitsyni* fossil taxon; minimum hard bound = 125.7 Mya, maximum soft bound = 521 Mya. Branchiopoda were dated using *Lepidocaris rhyniensis* fossil taxon; minimum hard bound = 407.6 Mya, maximum soft bound = 521 Mya.

Nucleotide composition was calculated with MEGA v.7. Variation of GC content between clades and ancestor was evaluated on the phylogenetic dataset (PCGs+rRNAs). Estimates of ancestral GC content were obtained by reconstructing ancestral sequences at Anostraca and Onychocaudata/Notostraca stem node using FastML web server [55] (available at http://fastml.tau.ac.il/, last accessed November 2018). The search was set as follows: PCGs+rRNA alignment were analyzed using the maximum-likelihood tree as guide tree, with GTR + G substitution model and branch lengths optimization. Following the recommendation of Matsumoto et al. [56], we sampled 100 ancestral sequences (Additional files 1 and 2) from the posterior distribution at the two selected stem nodes and obtained average GC values to compare with GC content of extant taxa.

Different substitution rates among clades were tested using the Two-Cluster test, implemented in LINTRE [57]. A tree file formatted for LINTRE was manually built using branch lengths from ML trees and used as input for the Two-Cluster test analysis.

Tests for positive selection were carried out by analyzing the dN/dS ratio (ω) over branches in the ML obtained on PCGs only, using the *codeml* algorithm implemented in PAML 4.8 [58]. We tested two different models: the one-ratio model, assuming a single ω value for the whole tree (*codeml* model = 0) and the free-ratios model, assuming a ω value for each branch (*codeml* model = 1). Finally, a likelihood-ratio test was used to calculate the best fitting model.

## Results and discussion

### Mitochondrial genome characterization

**Fig. 1:**
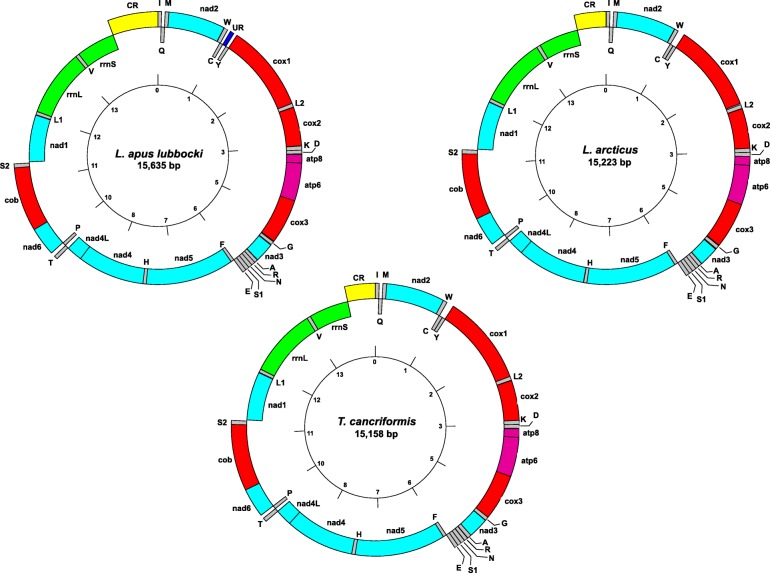
Schematic structures of newly sequenced mitochondrial genomes

Assembled mitochondrial genomes ranged in length from 15,158 bp (*T. cancriformis*) to 15,635 bp (*L. apus lubbocki*) (Table 2; Fig. 1). The overall mitogenome structure is congruent with the ancestral Pancrustacea model [10], showing 13 PCGs, two rRNA genes and 22 tRNA genes (Fig. 1; Additional file 3: Table S2).

**Table 2:** General information and nucleotide features of newly sequenced mitogenomes and of those drawn from Genbank

Species	Length (bp)	Mean coverage	GC-richness				
			Total	PCGs	rRNAs	tRNAs	Control Region
*Lepidurus apus lubbocki*	15,635	234×	27.8	28.6	27.2	28.5	15.7
*L. arcticus*	15,223	200×	32.5	33.8	27.9	29.8	27.5
*Triops cancriformis* (Italy)	15,158	234×	31.3	31.4	29.1	31.5	29.1
*T. cancriformis* (Spain)	15,103	295×	31.3	31.4	29.1	31.5	29.1
*T. cancriformis* (Japan)	15,101	–	31.3	31.3	29.6	31.5	28.1
*T. australiensis*	15,125	–	28.4	28.5	27.3	32.1	25.4
*T. longicaudatus 1*	15,110	–	30.7	31.2	28.5	30.3	26.7
*T. longicaudatus 2*	15,115	–	30.6	31.1	28.9	30.1	26.8
*T. longicaudatus “l”*	15,047	–	30.1	30.4	28.3	30.5	26.4
*T. longicaudatus “s”*	15,028	–	30.5	31.0	28.6	30.7	27.5
*T. newberry*	14,976	–	30.8	31.4	28.6	30.6	27.5
*Artemia franciscana*	15,822	–	35.6	35.9	37.3	34.8	31.9
*A. tibetiana 1*	15,826	–	37.3	37.6	38.7	35.4	34.5
*A. tibetiana 2*	15,742	–	37.3	37.8	38.6	35.4	34.6
*A. urmiana*	15,945	–	37.5	38.2	38.0	35.5	36.0
*Phallocryptus tserensodnomi*	16,493	–	34.6	36.2	33.2	33.9	29.4
*Streptocephalus sirindhornae*	16,887	–	35.4	35.6	33.4	32.5	37.4
*Limnadia lenticularis*	15,151	–	35.0	35.8	31.5	34.7	29.4
*Diaphanosoma dubium*	16,353	–	34.4	34.9	32.5	34.5	41.9
*Daphnia carinata*	15,245	–	29.7	30.1	27.9	29.6	27.4
*D. galeata*	16,160	–	36.3	38.2	31.7	34.0	32.0
*D. magna*	14,948	–	32.9	33.7	29.8	31.2	26.3
*D. pulex*	15,333	–	37.8	39.9	32.3	34.4	32.9
*D. pulex (China)*	15,306	–	35.5	36.7	31.6	33.8	34.6

The average GC content (Table 2) ranges from 27.8% (*L. apus lubbocki*) to 32.5% (*L. arcticus*). PCGs are more GC-rich than rRNA and tRNA genes only in *L. arcticus*; the control region of *L. apus lubbocki* is the most AT-rich region among the three taxa.

Nine out of the 13 PCGs (*atp6*, *atp8*, *cox1*, *cox2*, *cox3*, *cob*, *nad2*, *nad3*, *nad6*) are encoded on the H strand, while four are encoded on the L strand (*nad1*, *nad4*, *nad4L*, *nad5*). In *T. cancriformis*, most PCGs have ATG (*n* = 6) or ATA (*n* = 5) start codons; the *nad2* and the *nad5* genes use ATT and GTG start codons, respectively (Additional file 3: Table S2). The most represented stop codon is TAA, except for the *nad3* gene, with TAG, and *cox3*, *nad5* and *nad4* genes that have an incomplete TA- stop codon (Additional file 3: Table S2). The *Lepidurus* taxa have similar PCG features: seven genes use the ATG start codon, followed by the ATA (*n* = 5) and a TGA start codon (*nad1* gene) (Additional file 3: Table S2). Even in this instance, TAA is the most used stop codon; only two genes use a different stop codon: *nad4*, using the incomplete TA- codon, and *nad5*, using the TAG codon (Additional file 3: Table S1). The GC richness of PCGs varies from 28.6% (*L. apus lubbocki*) to 33.8% (*L. arcticus*) (Table 2).

The analysis of relative synonymous codon usage (RSCU) among the two *Lepidurus* taxa and *T. cancriformis* did not demonstrate substantial differences in codon usage, except for the leucine-encoding TTA in *L. apus lubbocki* (Additional file 4: Figure S1). All codons were present in PCGs of the examined mitogenomes, except the AGG codon that is absent in *L. apus lubbocki* and *T. cancriformis* taxa, and poorly represented in *L. arcticus* (Additional file 4: Figure S1).

The two rRNA genes, *rrnL* and *rrnS*, are encoded on the L strand and have GC content < 30.0% in all samples (Table 2). The length of *rrnS* is the same in the two *Lepidurus* taxa, 763 bp, and a little shorter in *T. cancriformis*, 756 bp. The *rrnL* gene, though, shows length variation also between *L. apus lubbocki* and *L. arcticus*, being 3 bp shorter in the latter species. *Triops cancriformis rrnL* gene is slightly shorter than the *Lepidurus* ones (Additional file 3: Table S2).

The tRNA gene lengths span between 63 bp and 73 bp, with an average GC richness between 28.5% (*L. apus lubbocki*) and 31.5% (*T. cancriformis*) (Table 2; Additional file 3: Table S2). All tRNAs show the typical cloverleaf structure, except the *trnS1* that lacks the D-arm; this is common among metazoan serine-tRNAs [59].

The control-region length in *Lepidurus* taxa varies between 545 bp (*L. arcticus*) and 885 bp (*L. apus lubbocki*), mainly owing to a composite microsatellite motif (CTATTTAT)_22_(TTTA)_18_(CTATTTAT)_9_(TTTA)_15_ in *L. apus lubbocki*. This repeat motif, moreover, appears to be responsible for the AT richness of this region (Table 2): in fact, if excluded from the analysis, the GC content rises to 21.2%. The length of the control region in the *T. cancriformis* samples is 467 bp (Spain) and 522 bp (Italy), the difference being due to a 55 bp duplication at the 5′ end of the latter.

**Fig. 2:**
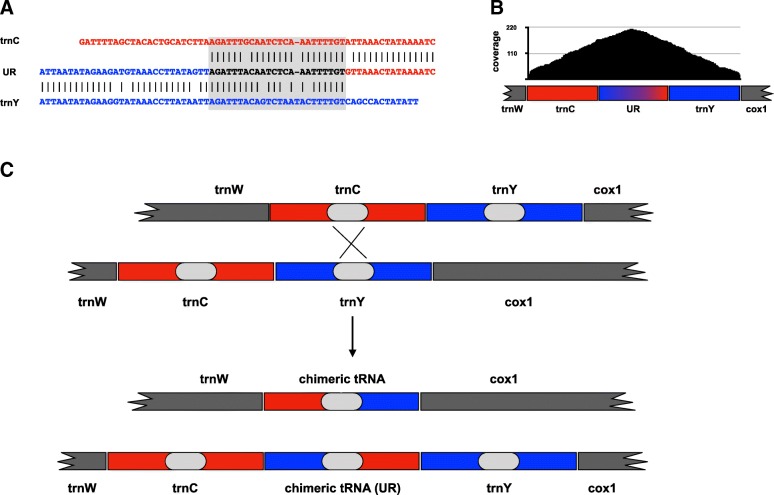
*Lepidurus apus lubbocki* unassigned region (UR) sequence analysis. **a** Alignment of UR with flanking tRNAs; the grey shaded area indicates the region of high similarity between *trnC* and *trnY*. **b** Coverage of the UR obtained by reads mapping. **c** Schematic drawing of the hypothesized non-homologous recombination originating the observed UR

Within the newly sequenced mitogenomes there are few intergenic regions, spanning from 1 to 22 nucleotides. On the other hand, a larger unassigned region (UR) of 69 bp can be observed in the *L. apus lubbocki* mitogenome, located between the *trnC* and *trnY* genes (Fig. 1; Additional file 3: Table S2). A visual inspection reveals that the UR is a chimeric sequence between the two flanking tRNAs; moreover, a high similarity region (82.6%) is shared between *trnC* and *trnY* sequences, corresponding to the anti-codon arm (Fig. 2a). In order to determine whether the chimeric structure derives from an assembly error, reads were mapped on the region spanning the *trnC* + UR + *trnY*: this showed a maximum coverage of 214×, with 109 reads covering entirely the whole UR (Fig. 2b), allowing us to rule out the possibility of an assembly artefact. Alternatively, it can be hypothesized that the assembled UR may have included reads from a nuclear mitochondrial DNA segment (NUMT) [60]. NUMTs are usually a serious issue in PCR-based approaches, because of preferential PCR amplification due to possible biases in primer annealing on NUMT sequence rather than on true mitochondrial DNA. However, this should not be an issue in whole genome sequencing. In addition, because of their high cellular copy number, mitochondrial reads are highly represented in genome sequencing, greatly outnumbering those of NUMTs [61]. Furthermore, NUMTs are pseudogenic sequences that can be typically distinguished by the accumulation of substitutions and indels with respect to genuine mitochondrial sequences [60]; therefore, we would have seen different pools of reads mapping to either the NUMT or the mitochondrial genome. Thus, we checked for the possible presence of a mtDNA variant without the UR and showing the structure expected from other notostracan mitogenomes. We manually produced a sequence which included only the *trnC* and *trnY* genes and used it to map reads: this resulted in no read mapping, thus excluding the possibility of having missed the ancestral variant and making unlikely that we mistakenly assembled a NUMT in the *L. apus lubbocki* mitogenome.

Overall, these results suggest the UR could be the product of a non-homologous recombination. It is, in fact, possible that two mitochondrial molecules mis-aligned due to the similarity of the anti-codon arm domain and an unequal DNA exchange occurred (Fig. 2c). Then, two different haplotypes were produced: one including only a chimeric tRNA but not the *trnC* and *trnY* genes, the other including a chimeric tRNA (the actual UR) and the two flanking tRNA genes (Fig. 2c). In order to check whether sequences including only the chimeric tRNA were present but not assembled, we manually created this sequence, and found that no reads map to it. We can conclude that the variant carrying only the chimeric tRNA is not present in the sequenced individual; since this variant would lack two tRNA genes, it is likely that it has been lost due to natural selection.

Homologous and non-homologous mitochondrial DNA recombination has been reported in animal mitochondria, both in vitro or in vivo [62]; in particular, evidence of non-homologous recombination has been reported in the control region of the root-knot nematode *Meloidogyne javanica* [63]. To our knowledge, data presented here constitute the first evidence of a possible non-homologous recombination event within the genic part of the mitochondrial genome. It is interesting to speculate about the fate of the observed UR. Although originating from the recombination of two tRNA genes, the observed UR was not recognized as a tRNA by the annotation software and it does not fold into a proper cloverleaf structure. Following the secondary structures of the flanking *trnC* and *trnY*, we sought to reconstruct a hypothetical secondary structure for the UR (Additional file 4: Figure S2). While generally consistent with the typical cloverleaf structure, with a conserved tyrosine anticodon, lack of complementarity between the two strands of the acceptor arm seems to preclude its functionality. It is possible that point mutations occurring in this domain could stabilize the structure, which opens the possibility of recruiting the UR as a new copy of *trnY* gene.

### Gene order variability among Branchiopoda

**Fig. 3:**
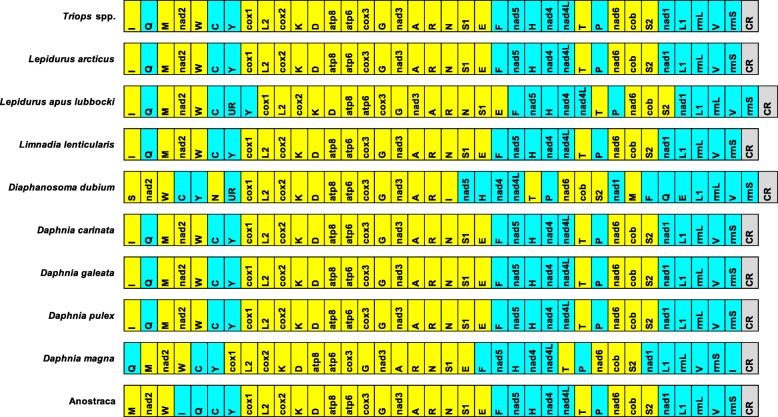
Gene arrangement in analyzed branchiopod mitogenomes. The yellow and cyan colours indicate genes on the H and L strand, respectively

With the exception of the UR in the *L. apus lubbocki*, notostracan mitogenomes share the same gene order, consistent with the ancestral Pancrustacea model (Fig. 3). Within Onychocaudata, the same holds also for *Limnadia lenticularis*, and most of *Daphnia* species. In the *Daphnia magna* mitogenome, on the other hand, the *trnI* gene and the control region experienced an inversion. The most rearranged mitogenome, actually, is that of *Diaphanosoma dubium* showing extensive rearrangement of tRNA genes. Moreover, it showed a 978 bp long UR, upstream the *cox1* gene: this appears to have a coding potential but the resulting protein (325 aa) does not match any known protein (not shown) [40]. Anostracan mitogenome does not vary among the five species analyzed, although it showed a tRNA rearrangement with respect to the ancestral Pancrustacea model (Fig. 3). Generally speaking, therefore, branchiopod mitogenomes appear well conserved, although showing a certain degree of structural variability.

### Nucleotide variability and phylogenetic analyses

The nucleotide divergence within genera observed among PCGs and rRNAs of newly sequenced mitogenomes ranged from 0.18%, in a comparison between Italian and Spanish *T. cancriformis*, to 20.25%, as scored between the two *Lepidurus* taxa. Moreover, with respect to the Japanese *T. cancriformis,* the Italian and Spanish *T. cancriformis* sequences diverge by 0.24 and 0.32%, respectively (Table 3). These estimates are in line with previous mitochondrial survey between *Triops cancriformis* populations and between *Lepidurus* species [22, 24]. An overall evaluation among Notostraca (Additional file 4: Figure S3) revealed that the least variable genes are the rRNAs (divergence *rrnL* = 17.3% and *rrnS* = 17.5), while the most variable ones resulted the *nad6* (37.6%) and the *atp8* (37.9%). On average, rRNA genes are the less variable and the genes encoding for NADH subunits are the most variable. This trend is maintained when all available branchiopod mitogenomes (*N* = 24) are considered, with the only difference that the least variable gene is *cox1* (Additional file 4: Figure S3).

**Table 3:** Sequence divergence (%) between Notostraca taxa based on PCG and rRNA gene sequences. Species with newly sequenced mitogenomes are marked in bold

	1	2	3	4	5	6	7	8	9	10
1. ***L. arcticus***										
2. ***L. apus lubbocki***	20.25									
3. ***T. cancriformis*** (Italy)	26.77	26.13								
4. ***T. cancriformis*** (Spain)	26.80	26.19	0.18							
5. *T. cancriformis* (Japan)	26.78	26.17	0.24	0.32						
6. *T. longicaudatus* “l”	26.16	25.66	22.06	22.11	22.09					
7. *T. longicaudatus* “s”	26.03	25.55	22.04	22.09	22.06	3.84				
8. *T. longicaudatus* 1	26.23	25.74	22.26	22.30	22.25	4.08	2.04			
9. *T. longicaudatus* 2	26.12	25.69	22.14	22.16	22.14	4.16	1.73	2.54		
10. *T. newberry*	26.12	25.66	22.03	22.08	22.03	3.75	1.83	0.72	2.17	
11. *T. australiensis*	25.50	24.73	22.02	22.08	22.03	15.37	15.39	15.57	15.29	15.33

**Fig. 4:**
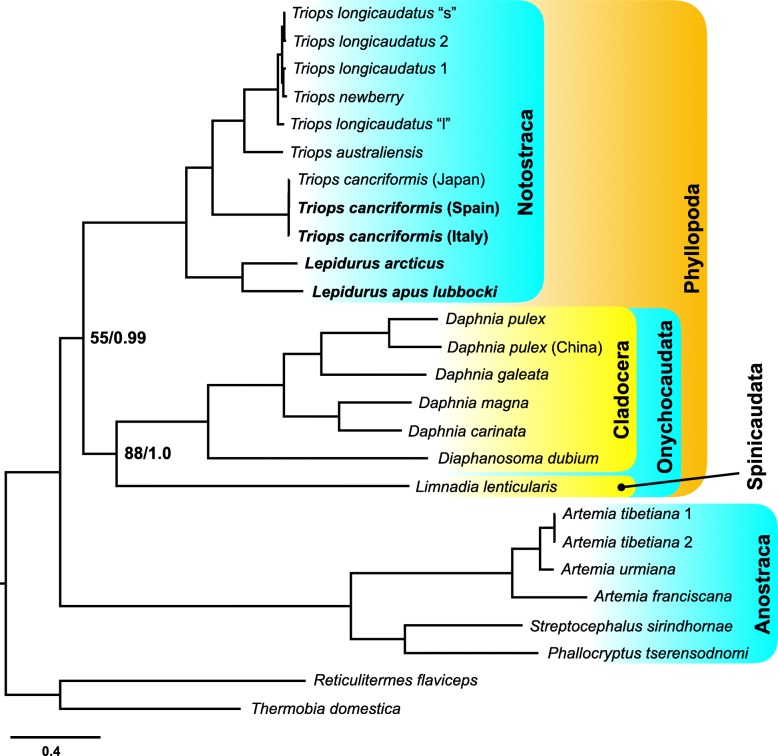
Schematic drawings of Maximum-likelihood (*−lnL* = 196,010.77) and Bayesian inference (*−lnL* = 194,237.10) phylogenetic analyses. All nodes received maximum bootstrap and posterior probability support, unless differently indicated. Newly sequenced mitogenomes are indicated in bold

Phylogenetic analyses were conducted on concatenated PCG and rRNA genes. The whole dataset consisted of 14,274 aligned nucleotide positions, with 10,214 variable and 9266 parsimony informative sites. Both maximum-likelihood analysis and the Bayesian inference gave a topology (Fig. 4) in agreement with the current knowledge on branchiopod phylogeny [4, 6–8]. The deepest node splits the two clusters representing the Anostraca and the Phyllopoda clades. The two groups are well supported in the Bayesian inference, showing posterior probability equals to 1.0 and 0.99, respectively, but in the maximum-likelihood tree the Phyllopoda clade is only weakly supported (bootstrap = 55%). Within Anostraca, the split between suborders Artemiina (*Artemia* spp.) and Anostracina (*Phallocryptus* + *Streptocephalus*) had the maximum support in both elaborations. Within Phyllopoda, Onychocaudata (*Limnadia lenticularis* + cladoceran taxa) received maximum nodal support only in the Bayesian inference, while Notostraca is fully supported in all analyses. Relationships within Notostraca fully agree with previous analyses: the two *Lepidurus* sequences cluster together and are in sister relationship with the *Triops* clade [64, 65].

### Nucleotide compositional bias among Branchiopoda

Branchiopod mitogenomes are generally AT-rich, the GC content varying between 28.2% in *T. australiensis* to 38.9% in *D. pulex* (Table 2). In the phylogenetic dataset, the average GC content was 30.5% in Notostraca, 35.0% in Onychocaudata and 36.8% in Anostraca, and results significantly different between clades (Kruskal-Wallis *P* < 0.001). Pairwise comparisons indicate that Notostraca have a significantly lower GC content than Anostraca or Onychocaudata (Mann-Whitney pairwise post-hoc test, with sequential Bonferroni correction, *P* < 0.01), while the latter two clades do not differ significantly (Mann-Whitney post-hoc test *P* = 0.197). In order to determine whether nucleotide composition has varied since the common ancestor, which would mean a shift in nucleotide composition during the evolution of the three clades, we reconstructed the ancestral sequences at stem nodes of each of the three orders and compared their GC content to that of extant lineages. To avoid possible biases in the reconstruction process we followed the recommendation in Matsumoto et al. [56], sampling multiple sequences from the posterior distribution (*n* = 100; Additional files 1 and 2) and calculated the average GC content. Average GC content of ancestral sequences sampled from the posterior distribution at Anostraca stem node is 31.0%, very close to the 30.5% calculated at Onychocaudata and Notostraca stem nodes. Overall, GC content varies significantly among ancestral nodes and extant lineages (Kruskal-Wallis *P* < 0.001); however, while Notostraca do not show a significant departure from ancestral GC content (Mann-Whitney post-hoc test *P* = 0.249), Anostraca and Onychocaudata sequences show a significant increase of GC richness from their relative stem node (Mann-Whitney post-hoc test *P* < 0.001 and *P* < 0.01, respectively). Therefore, the observed pattern of nucleotide composition in Branchiopoda can be explained by a preferential AT to GC substitution bias during the evolution of Anostraca and Onychocaudata lineages. Differential GC contents and inferred substitution bias do not appear to have had substantial effects on phylogenetic reconstructions, although it cannot be excluded that the weak support at Phyllopoda clade could be due to similar GC content between Anostraca and Onychocaudata mitogenomes.

### Differential substitution rates between clades

In all phylogenetic elaborations, the branches of Onychocaudata and Anostraca clades, including stem branches, appeared longer than those of the Notostraca clade (Fig. 4). Interestingly, the same pattern can be observed in a recently published phylogenomic study (> 600 orthologues) [8]. Substitution rate constancy throughout the tree was rejected (likelihood-ratio test, ∆_*lnL*_ = 55,050,314.81; *P* < 0.0001), and the two-cluster test analysis (Additional file 3: Table S3) clearly indicates that, with respect to the Phyllopoda cluster, Anostraca show a significantly higher substitution rate (∆ = 0.0462 ± 0.0018; *Z* = 25.42, confidence probability = 99.96%). Furthermore, Onychocaudata show a higher substitution rate with respect to Notostraca (∆ = 0.0258 ± 0.0011; *Z* = 22.18, confidence probability = 99.96%). Therefore, these data clearly support a differential substitution rate of Notostraca with respect to the Onychocaudata and Anostraca clades.

**Fig. 5:**
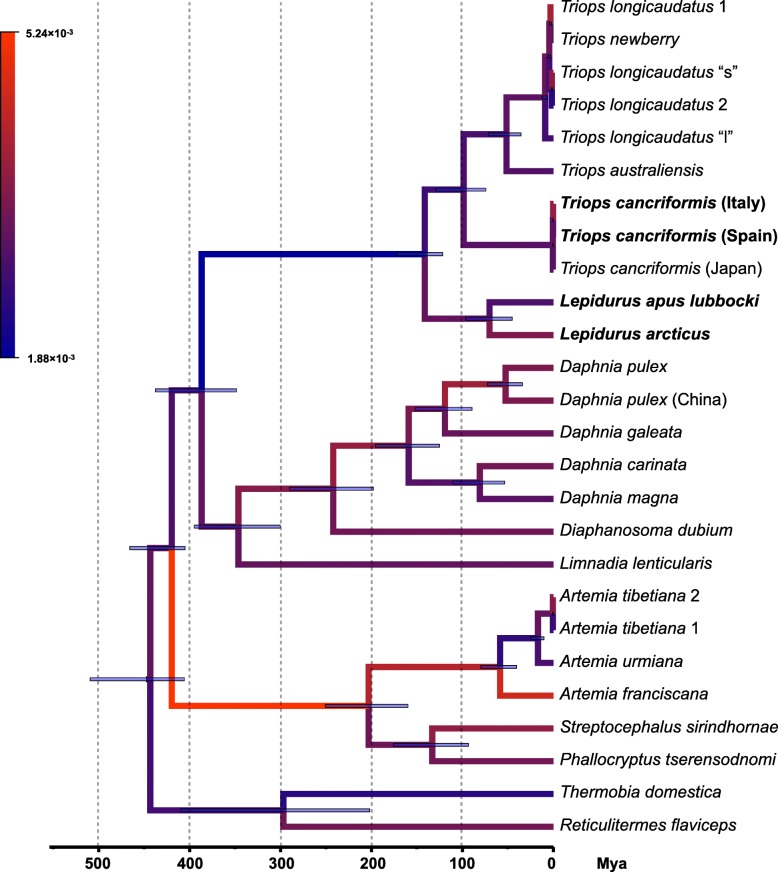
Time calibrated Bayesian tree. All nodes received maximum posterior probability support. Node bars represent 95% high posterior density of age estimate. Branch colours vary accordingly to substitution rate (substitutions/site/million year), as indicated in the upper-left legend. Newly sequenced mitogenomes are indicated in bold

In order to calculate the substitution rate per branch, a calibrated time tree was built by dating Notostraca, Cladocera and Anostraca crown groups, along with the tree root, using well-detailed and justified fossil records described in Wolfe et al. [54] for points calibration. The obtained tree topology resulted identical to those produced with maximum-likelihood and Bayesian inference elaborations, with all nodes receiving maximum posterior probability support (Fig. 5). Inferred age estimates reconcile well with those obtained in previous analyses [64, 65]: diversification of Branchiopoda occurred since 425.7 Mya and extant lineages of Notostraca, Cladocera, and Anostraca (crown groups) radiated between 243.0 Mya and 143.9 Mya. The estimated mean substitution rate is 3.21 × 10^− 3^ substitutions/site/million year (95% high posterior density interval = 2.81 × 10^− 3^, 3.55 × 10^− 3^). This rate varies among branches between 1.88 × 10^− 3^ substitutions/site/million year and 5.24 × 10^− 3^ substitutions/site/million year, the two values being estimated for Notostraca and Anostraca stem branches, respectively. Overall, it seems that substantial rate variation occurred along the stem branches of analyzed clades (Fig. 5; Additional file 4: Figure S4). The lower substitution rate observed along the Notostraca stem branch, 2.7-fold and 1.7-fold with respect to Anostraca and Diplostraca (1.9-fold vs Cladocera), respectively, could be either interpreted as i) an episodic substitution rate decrease along the Notostraca stem branch, or ii) an acceleration of the substitution rate in the other stem lineages with respect to the notostracan one.

These data are in agreement with those from a recent genome-wide survey where the nucleotide substitution rate calculated for *L. arcticus* and *L. apus lubbocki* was found to be significantly lower than the one calculated for *D. magna* and *D. pulex*. In contrast to this, however, the amino acid substitution rate did not vary significantly, in line with the observed general pattern of negative selection [25]. We therefore checked the extent of selective pressures on mitogenomic sequences calculating the ω ratio across the tree branches. Two different models were run: the *free-ratio* model, allowing an ω value for each branch and the *one-ratio* model, considering a single ratio for the whole tree. The *free-ratio* model resulted the best fit one (likelihood-ratio test, ∆_*lnL*_ = 608.002, *P* < 0.001). No instances of positive selection, ω > 1, have been found; the highest ω values were scored on the Phyllopoda (0.493) and Onychocaudata (0.402) stem branches, possibly suggesting a relaxation of natural selection on these branches. Interestingly, both Anostraca and Notostraca stem branches show decidedly lower ω values (0.127 and 0.184, respectively), consistent with purifying selection. Overall, the present data do not support any correlation between the observed substitution rates variation and changes in natural selection regime, in agreement with genome-wide data [25].

Variation in substitution rates could depend not only on life-history traits, such as generation time, lifespan, and body size, but also on speciation rate [66–70]. In Branchiopoda, it has also been suggested that halophilic habits may correlate with faster substitution rates [71]. Although these hypotheses cannot be reliably tested with our species sampling, an alternative explanation could be linked to the nucleotide substitution bias observed among the three branchiopod orders. Based on mathematical models and simulations, Sueoka [72] showed that a directional mutation bias during lineages’ evolution may significantly affect substitution rates. Accordingly, an episodic acceleration of substitution rate along the Diptera stem branch has been observed and found consistent with directional mutational bias during the evolution of this taxon [73]. Based on the conclusions of these studies, the observed GC-biased mutation rate would speak in favour of an increase of the substitution rate in Anostraca and Onychocaudata rather than an episodic deceleration in Notostraca, whose GC content appeared similar to that of the ancestor. Moreover, the Sueoka’s model [72] also predicts that changes in nucleotide composition would result in asymmetrical branch length in the phylogenetic tree, which fits with the phylogenetic tree obtained in our analysis.

## Conclusions

In the present study, we present four new Notostraca mitochondrial genomes, with two of them reported for the first time. We also carried out a comparative analysis with other available branchiopod mitogenomes. The analysis of mitogenomes’ gene order revealed a certain plasticity within and among Branchiopoda orders, and with respect to the ancestral Pancrustacea model. Notably, we detected the results of a possible unequal recombination in the *L. apus lubbocki* mitogenome which led to the formation of a pseudogenic tRNA variant. This is, to our knowledge, the first example of an unequal recombination event involving mitochondrial genes. Overall, the phylogenetic signal contained in the sequences appears reliable despite significant differences in substitution rate and nucleotide composition. The tree topologies here obtained confirm those previously obtained on fewer molecular markers or on phylogenomic datasets. In addition, we showed differential substitution rates among Branchiopoda orders; these could be likely linked to changes in nucleotide composition during evolution. As genomic data are accumulating, and rate differences have been observed also at the nuclear genomic level [25], it will be interesting to test this observation on a wider phylogenomic dataset.

## Additional files

Additional file 1: 100 ancestral sequences sampled from the posterior distribution of Anostraca stem node (FASTA 1394 kb)

Additional file 2: 100 ancestral sequences sampled from the posterior distribution of Phyllopoda stem node (FASTA 1394 kb)

Additional file 3: Supplementary Tables S1, S2, S3 (XLSX 18 kb)

Additional file 4: Supplementary Figures S1, S2, S3, S4 (PDF 218 kb)

## Data Availability

Newly obtained mitogenomes have submitted to GenBank under accession numbers MK579380-MK579383.
