# Supplementary material for: Mitochondrial genome diversity and evolution in Branchiopoda (Crustacea)
Source: Zoological Lett. 2019 May 27;5:15. doi: 10.1186/s40851-019-0131-5 (PMC6537178; doi:10.1186/s40851-019-0131-5)
Supplement: Supplementary file 4 — Supplementary Figures S1, S2, S3, S4 (PDF 218 kb) [file 40851_2019_131_MOESM4_ESM.pdf]

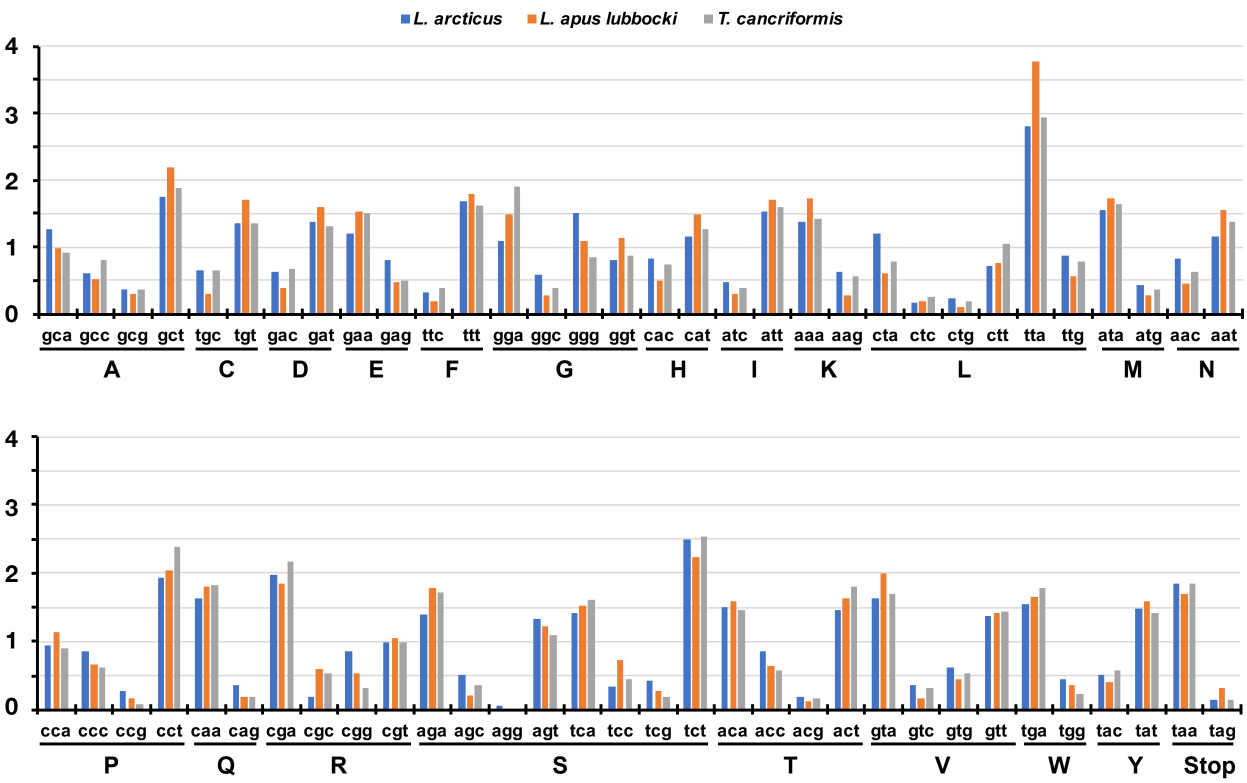

Fig. S1. RSCU analysis for the mitochondrial protein coding genes of the three analyzed Notostraca species.

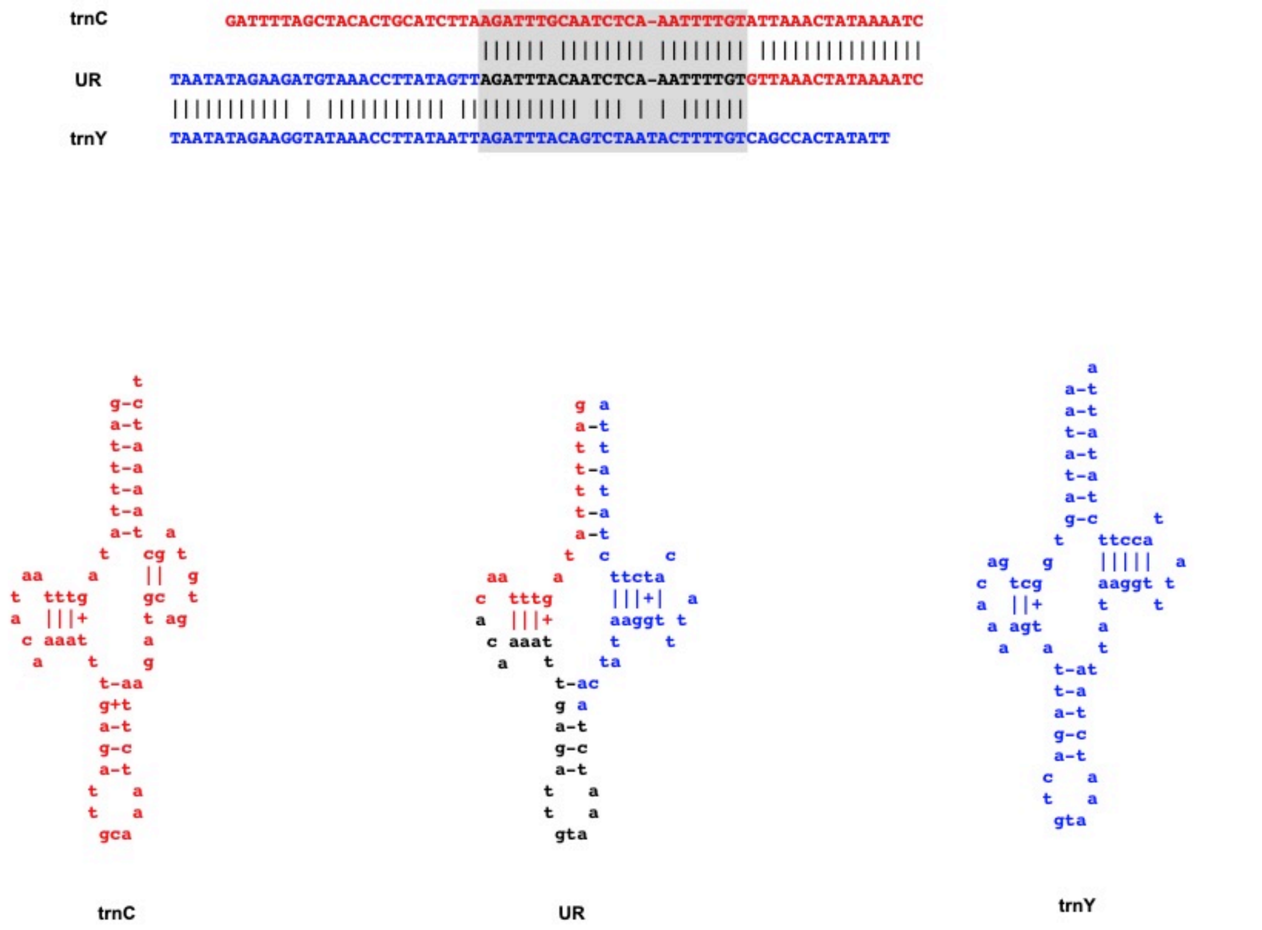

Fig. S2. Upper panel: Alignment of UR and flanking tRNAs (trnC and trnY). The gray area indicated the region of high similarity between tRNAs. Lower panel: clover leaf structures of tRNAs and the putative secondary structure for the UR.

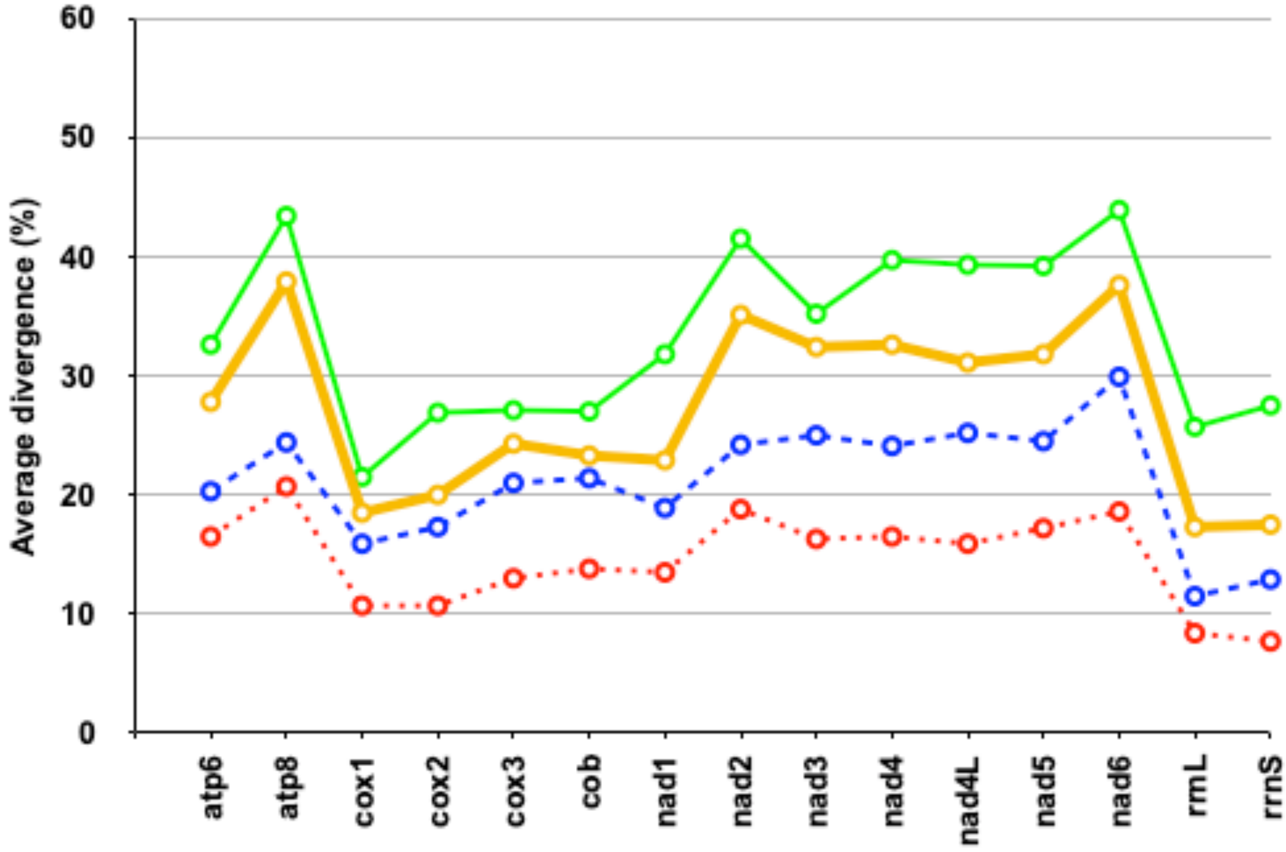

Fig. S3. Average nucleotide divergence (uncorrected p-distance) of protein coding and rRNA genes among i) *Triops* species (red, dotted line), ii) *Lepidurus* species (blue, dashed line), iii) Notostraca species (yellow line) and iv) Branchiopoda species (green line).

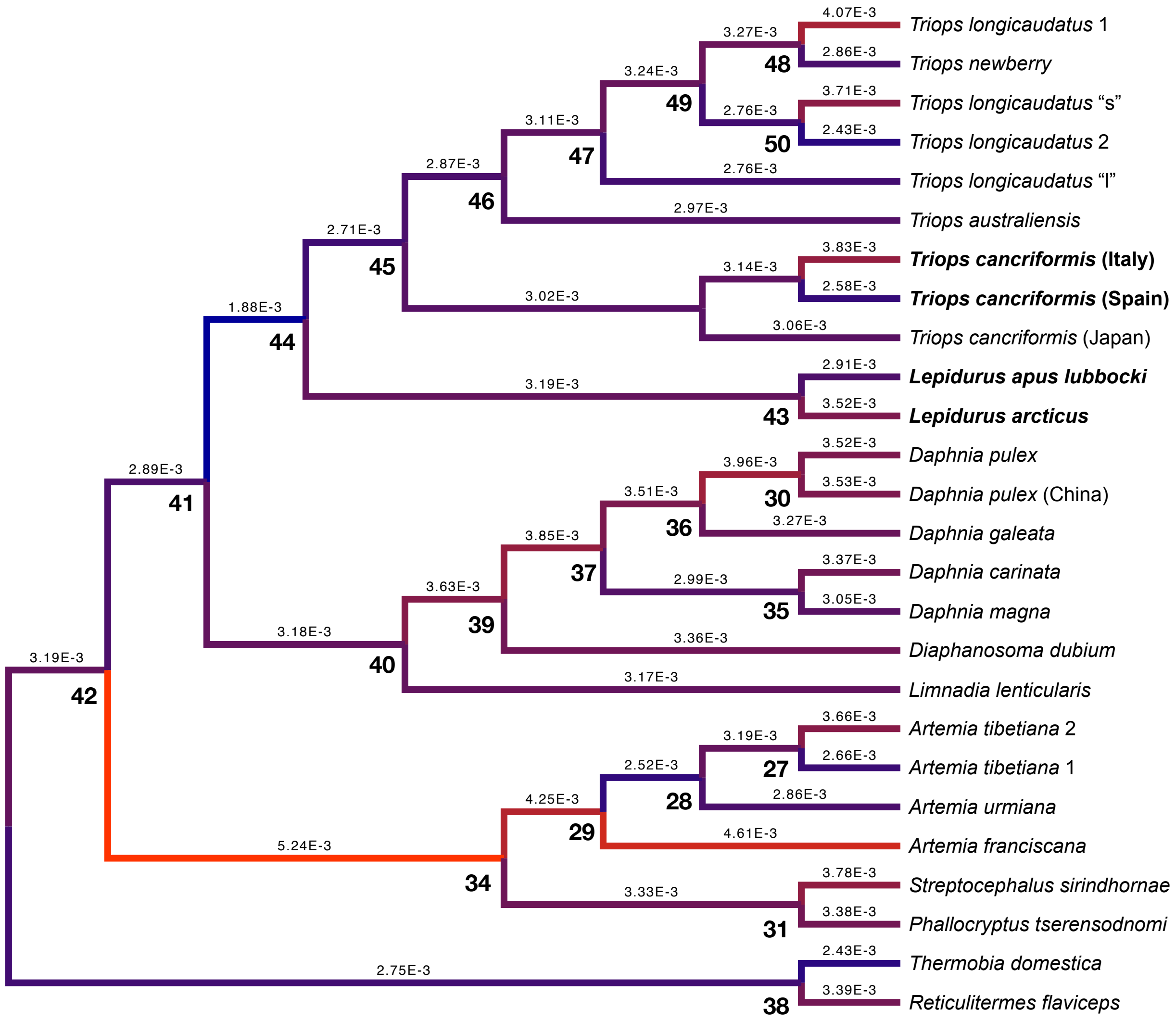

Fig. S4. Time calibrated Bayesian cladogram, with the number of substitutions/site/million year reported on branches. Branch colors varies with substitution rate, as in Figure 3. Newly sequenced mitogenomes are indicated in bold. Numbers at nodes are relative to Suppl. Table S3 (Additional file 1).
